# Case Report: Primary squamous cell carcinoma of the pancreas in a 75-year-old woman with a marked response to chemoimmunotherapy and conversion surgery

**DOI:** 10.3389/fmed.2026.1990719

**Published:** 2026-10-21

**Authors:** Xiaokun Hu, Yue Shi, Qiuyu Yu, Qingqing Zhu

**Affiliations:** 1 Department of Radiology, Sir Run Run Shaw Hospital, School of Medicine, Zhejiang University, Hangzhou, China; 2 Department of Pathology, Sir Run Run Shaw Hospital, School of Medicine, Zhejiang University, Hangzhou, China

**Keywords:** antineoplastic combined chemotherapy protocols, carcinoma, squamous cell, diagnostic imaging, immunotherapy, pancreatic neoplasms

## Abstract

Primary squamous cell carcinoma (SCC) of the pancreas is an extremely rare malignancy. Herein, we report a case of poorly differentiated primary pancreatic SCC in a 75-year-old woman. Computed tomography revealed a solid mass with cystic degeneration in the pancreatic head and neck with extensive central necrosis, abutment of the celiac axis and encasement of the main portal vein, while diffusion-weighted magnetic resonance imaging demonstrated restricted diffusion in the solid component. After multidisciplinary consensus, the patient received off-label nab-paclitaxel and gemcitabine combined with the PD-1 inhibitor serplulimab. After three cycles, serum CA19-9 decreased from 40.4 U/mL at baseline to 16.04 U/mL (reference range, < 37 U/mL) and the tumor regressed markedly on imaging. Subsequently, pancreaticoduodenectomy with segmental portal vein resection and reconstruction was successfully performed, achieving R0 resection without lymph node metastasis. Histopathology confirmed poorly differentiated SCC. No recurrence or metastasis was detected during five months of postoperative follow-up. In this report, we aim to provide imaging details that may facilitate recognition of this rare pancreatic malignancy and suggest that PD-1-based chemoimmunotherapy merits prospective evaluation as a conversion strategy in locally advanced disease.

## Introduction

Primary squamous cell carcinoma (SCC) of the pancreas is an extremely rare malignancy, accounting for less than 1% of pancreatic cancers. Because normal pancreatic tissue lacks squamous epithelium, its histogenesis remains uncertain, with several mechanisms proposed, including malignant transformation of squamous metaplasia, differentiation of pluripotent progenitor cells, and squamous transformation of pre-existing adenocarcinoma (1, 2). Diagnosis is challenging and requires exclusion of metastatic SCC from other primary sites. Owing to its rarity, no standard treatment strategy has been established, and the prognosis is generally poor. In a National Cancer Database study of 515 patients, the median overall survival was only 4 months, although patients undergoing surgical resection achieved substantially longer survival (1).

Immune checkpoint inhibitors have shown limited efficacy in conventional pancreatic ductal adenocarcinoma but are increasingly being explored in combination strategies. A recent phase II study demonstrated encouraging activity of serplulimab combined with gemcitabine, nab-paclitaxel, and stereotactic body radiotherapy in metastatic pancreatic ductal adenocarcinoma (3). Although PD-1 blockade combined with chemotherapy has previously achieved a marked response in locally advanced pancreatic SCC, successful conversion to radical resection has not, to our knowledge, been reported. Herein, we report a case of initially unresectable primary pancreatic SCC successfully converted to R0 resection after gemcitabine/nab-paclitaxel plus serplulimab, and describe its imaging, pathological, and therapeutic features. We present the following case in accordance with the CARE guideline.

## Case description

A 75-year-old Han Chinese woman presented with dull epigastric pain of two weeks' duration. The pain had no radiation, and she had no jaundice, fever or vomiting. Magnetic resonance imaging (MRI) arranged at a local hospital had shown a pancreatic head mass that was read as a possible cystadenoma with malignant transformation not excluded, and she was referred to our center. Her medical history included hypertension, coronary artery disease and cholecystectomy for gallstones; she did not have diabetes or chronic pancreatitis. She did not smoke or drink alcohol, and her family history was negative for malignancy. On admission she was afebrile, with a blood pressure of 128/62 mmHg; there was no scleral icterus, palpable lymphadenopathy, abdominal tenderness or palpable mass.

Routine haematological and biochemical tests were unremarkable. Serum CA19-9 was mildly raised at 40.4 U/mL (reference < 37 U/mL) and CA125 at 43.5 U/mL, whereas carcinoembryonic antigen (CEA) was normal at 3.3 ng/mL; SCC antigen was not measured, because SCC was not considered before treatment. CA19-9 fell to 28.58 U/mL after the first neoadjuvant cycle and to 16.04 U/mL after the second, and was 15.79 U/mL after surgery, while CA125 rose to 82.5 U/mL after surgery without any radiological evidence of disease.

Multiphasic contrast-enhanced computed tomography (CT) (SOMATOM Definition Flash, Siemens; 120 kVp, 270 mAs; 90 mL of iomeprol 400 mgI/mL at 3.5 mL/s) showed a lobulated cystic-solid mass, 63 mm × 34 mm × 39 mm, in the pancreatic head and neck with infiltrative margins. The solid component was iso- to hypodense on unenhanced images (about 32 HU vs. 37 HU in normal parenchyma) and enhanced peripherally in the arterial phase (about 108 HU), with persistent enhancement in the portal venous phase (about 117 HU); central necrosis or cystic change occupied roughly 70% of the tumor. The mass abutted the celiac axis with contact to the common hepatic and splenic arteries, encased the main portal vein, and narrowed the splenic vein-portal confluence, while the superior mesenteric artery was clear. The main pancreatic duct was mildly dilated in the body and tail. No regional lymphadenopathy, distant metastasis or ascites was present (Figures 1A–D). After three neoadjuvant cycles, follow-up CT showed slight tumor shrinkage with reduced enhancement (Figure 1E).

**Figure 1:**
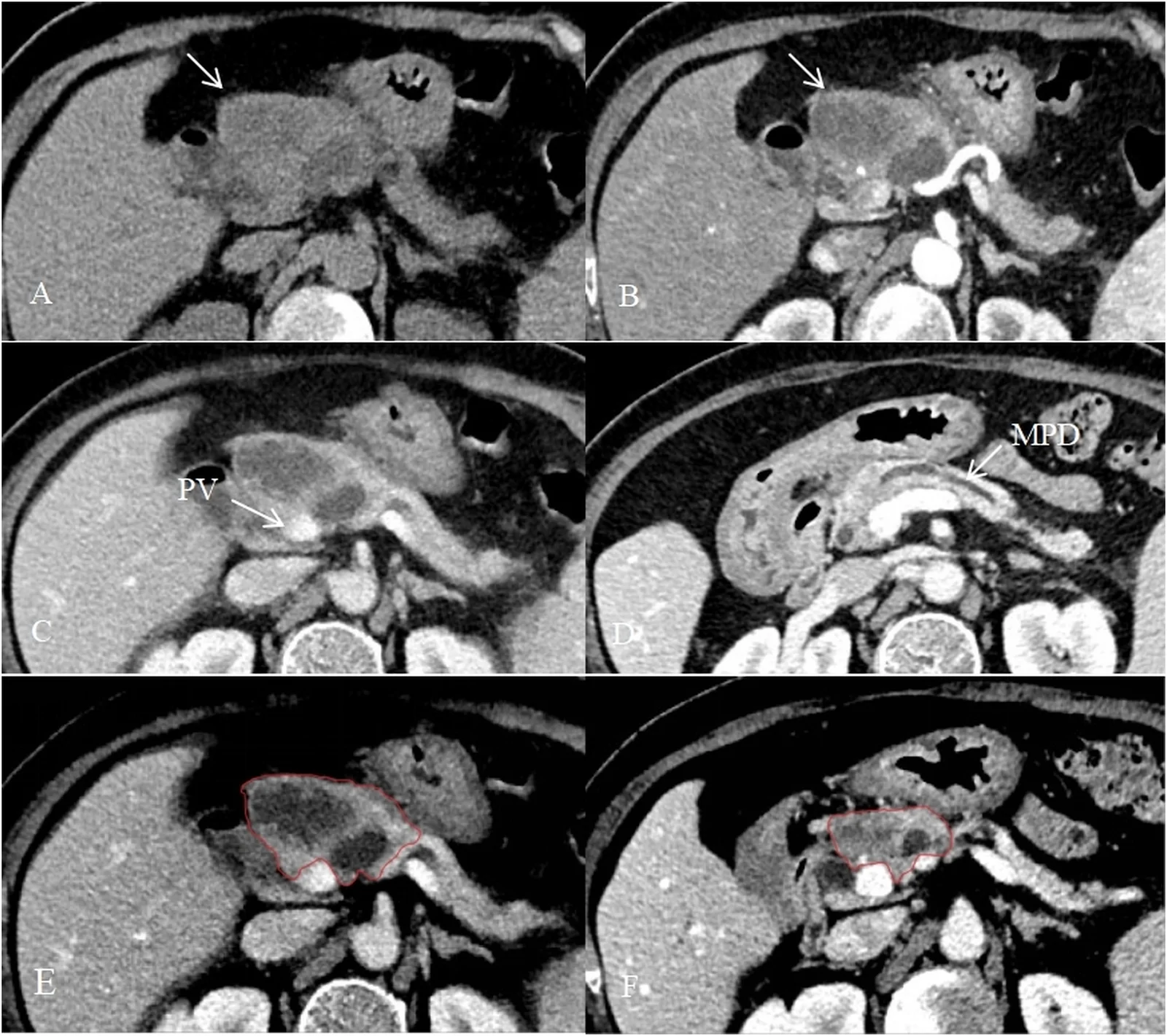
Preoperative multiphasic contrast-enhanced CT. **(A)** Unenhanced image shows an iso- to hypodense cystic-solid mass in the pancreatic head and neck (arrow); the solid component measures approximately 32 HU. **(B)** Arterial phase shows moderate peripheral enhancement of the solid component (arrow, approximately 108 HU) with a large central area of low-density necrosis. **(C)** Portal venous phase shows persistent enhancement (approximately 117 HU); the main portal vein (arrow) is encased by the tumor. **(D)** Portal venous phase shows mild dilation of the main pancreatic duct (arrow). **(E)** Arterial phase before treatment: the pancreatic head–neck mass (red circle) with peripheral enhancement. **(F)** After three cycles of AG plus serplulimab: the tumor is smaller (red circle) with slightly reduced enhancement, indicating treatment response. HU = Hounsfield units;PV = portal vein; MPD = main pancreatic duct.

MRI on a 3.0-T system (Signa HDxt, GE Healthcare) showed a solid component slightly hyperintense on T1-weighted images and hypointense and heterogeneous on T2-weighted images, surrounding markedly T2-hyperintense cystic areas. The solid component was hyperintense on diffusion-weighted imaging (b = 800 s/mm2) with corresponding hypointensity on the apparent diffusion coefficient (ADC) map; the measured ADC was 0.871 x 10_-3_mm_2_/s, clearly lower than that of normal pancreatic parenchyma (1.173 x 10_-3mm2/s_). After contrast injection the solid component enhanced less than the parenchyma in the arterial phase and filled in progressively on later phases (Figure 2). Endoscopic ultrasonography showed a hypoechoic mass with internal septa. EUS-guided fine-needle aspiration (EUS-FNA) was performed before neoadjuvant therapy and yielded poorly cohesive malignant cells reported as consistent with a malignant neoplasm. Squamous differentiation was not identified and no cell block suitable for immunohistochemistry was obtained, so the diagnosis of SCC was established only on the resected specimen.

**Figure 2:**
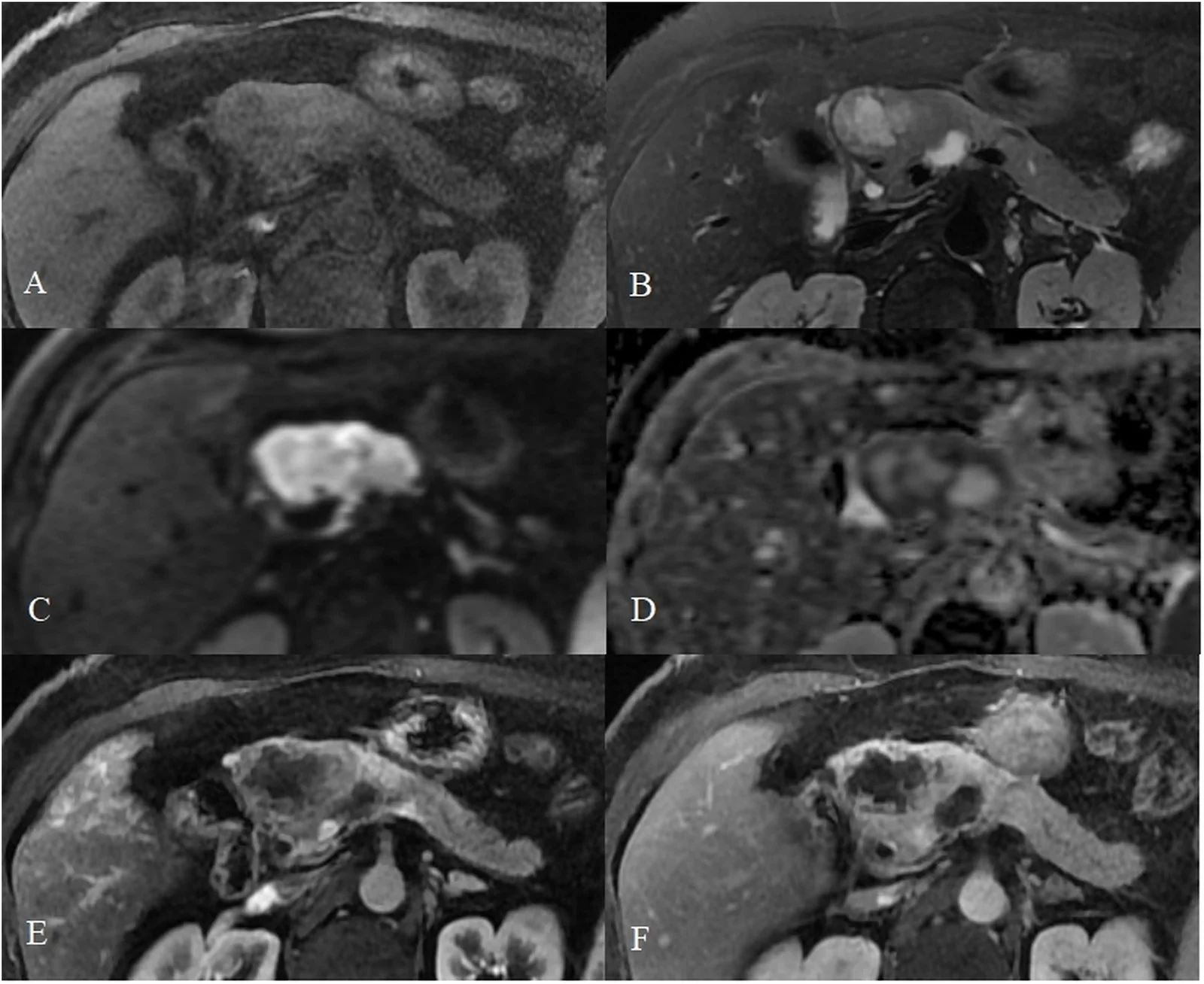
Preoperative magnetic resonance imaging (MRI). **(A)** T1-weighted image: solid component slightly hyperintense relative to the pancreas. **(B)** T2-weighted image: markedly hyperintense cystic areas with a hypointense, heterogeneous solid component. **(C)** Diffusion-weighted image (b = 800 s/mm2): hyperintense solid component. **(D)** Apparent diffusion coefficient (ADC) map: hypointense solid component; measured ADC 0.871 x 10_-3_mm_2_/s versus 1.173 x 10_-3_mm_2_/s in normal parenchyma. **(E,F)** Dynamic contrast-enhanced images show progressive enhancement of the solid component.

A multidisciplinary team regarded the tumor as locally advanced and initially unresectable because of portal vein encasement and arterial contact, and planned induction therapy followed by reassessment for resection. Gemcitabine plus nab-paclitaxel (AG) was chosen as the backbone because it is recommended for borderline-resectable and locally advanced pancreatic ductal adenocarcinoma in both the NCCN and CSCO guidelines. Adding serplulimab in this non-metastatic, unresectable setting is not part of any guideline recommendation and was given off-label after multidisciplinary consensus and detailed discussion with the patient, who provided written informed consent. The patient was not enrolled in a clinical trial. She received three 21-day cycles of nab-paclitaxel 120 mg and gemcitabine 1,000 mg on days 1 and 8 together with serplulimab 300 mg on day 1. All three cycles were completed as scheduled without dose reduction or grade 3–4 toxicity.

Pancreaticoduodenectomy with segmental portal vein resection and end-to-end anastomosis was then performed, with an estimated blood loss of 1,200 mL and an operating time of 510 min. The postoperative course was uneventful, with no pancreatic fistula, hemorrhage or infection. Pathology of the specimen showed a 4.9 cm × 2.0 cm × 2.0 cm tumor infiltrating the peripancreatic fat with R0 margins. The tumor was composed of sheets and nests of cells with necrosis, cholesterol clefts, keratin pearls and foci of poor differentiation, accompanied by a dense lymphoplasmacytic infiltrate; the cells had moderately eosinophilic cytoplasm, round nuclei, distinct membranes and prominent nucleoli. Immunohistochemistry was positive for p40 and p63 and negative for CK7, synaptophysin and chromogranin A, consistent with a poorly differentiated primary SCC (Figure 3). None of the 28 resected lymph nodes contained metastasis (peripancreatic 0/15, perienteric 0/7, periduodenal 0/2, and 0/4 along the superior mesenteric artery).

**Figure 3:**
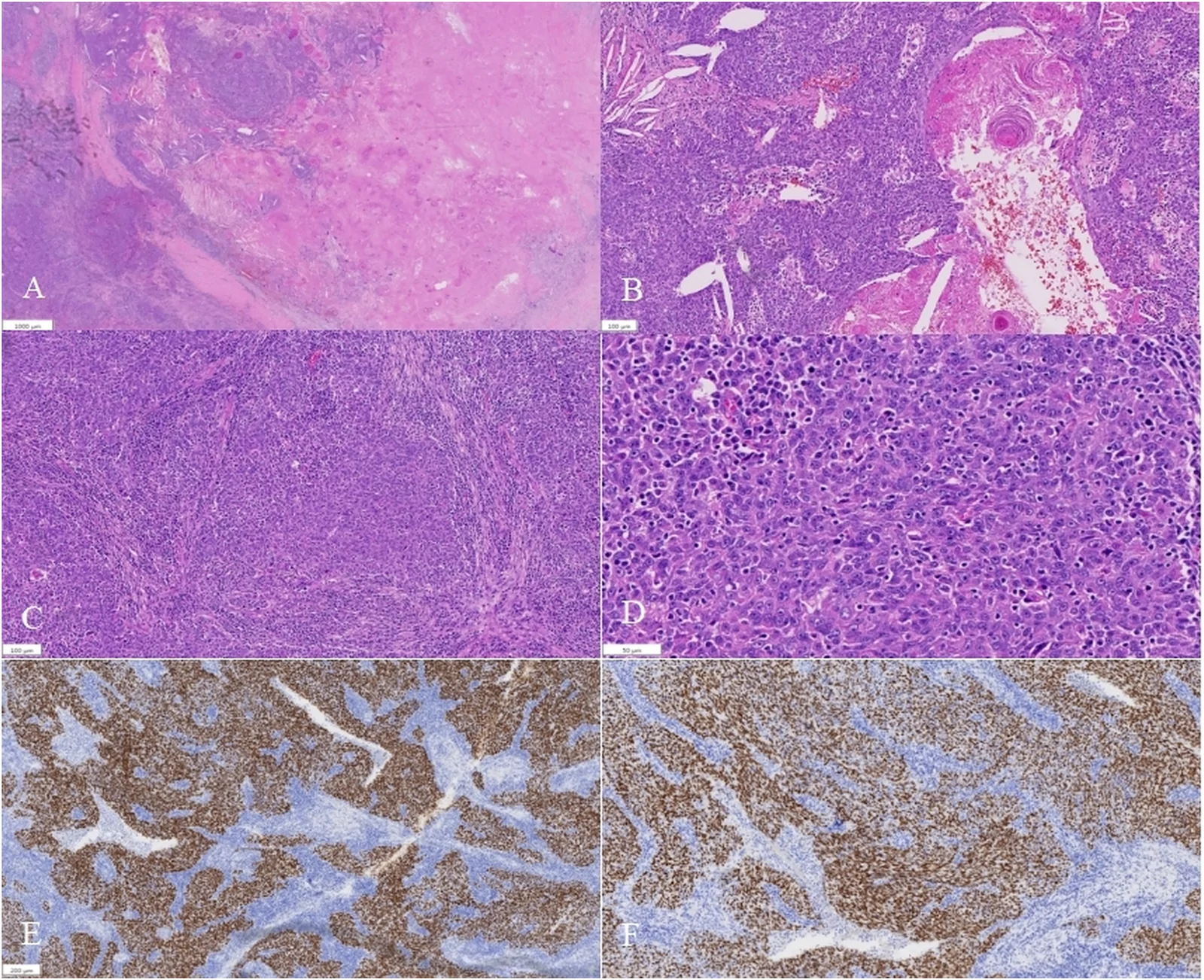
Histopathology and immunohistochemistry of the resected specimen. **(A–D)** Hematoxylin-eosin staining shows sheets and nests of tumor cells with necrosis, cholesterol clefts and keratin pearls, foci of poor differentiation with a dense lymphoplasmacytic infiltrate, and cells with moderately eosinophilic cytoplasm, round nuclei, distinct membranes and prominent nucleoli. **(E)** p40 immunostaining shows strong nuclear positivity. **(F)** p63 immunostaining shows strong nuclear positivity.

Follow-up was conducted from April 2 to September 2, 2026, through monthly outpatient visits and serial CT. No recurrence or metastasis was detected, CA19-9 stayed in the normal range, and the patient remained well. The diagnostic and treatment timeline, together with the dynamics of serum CA19-9, is shown in Figure 4.

**Figure 4:**
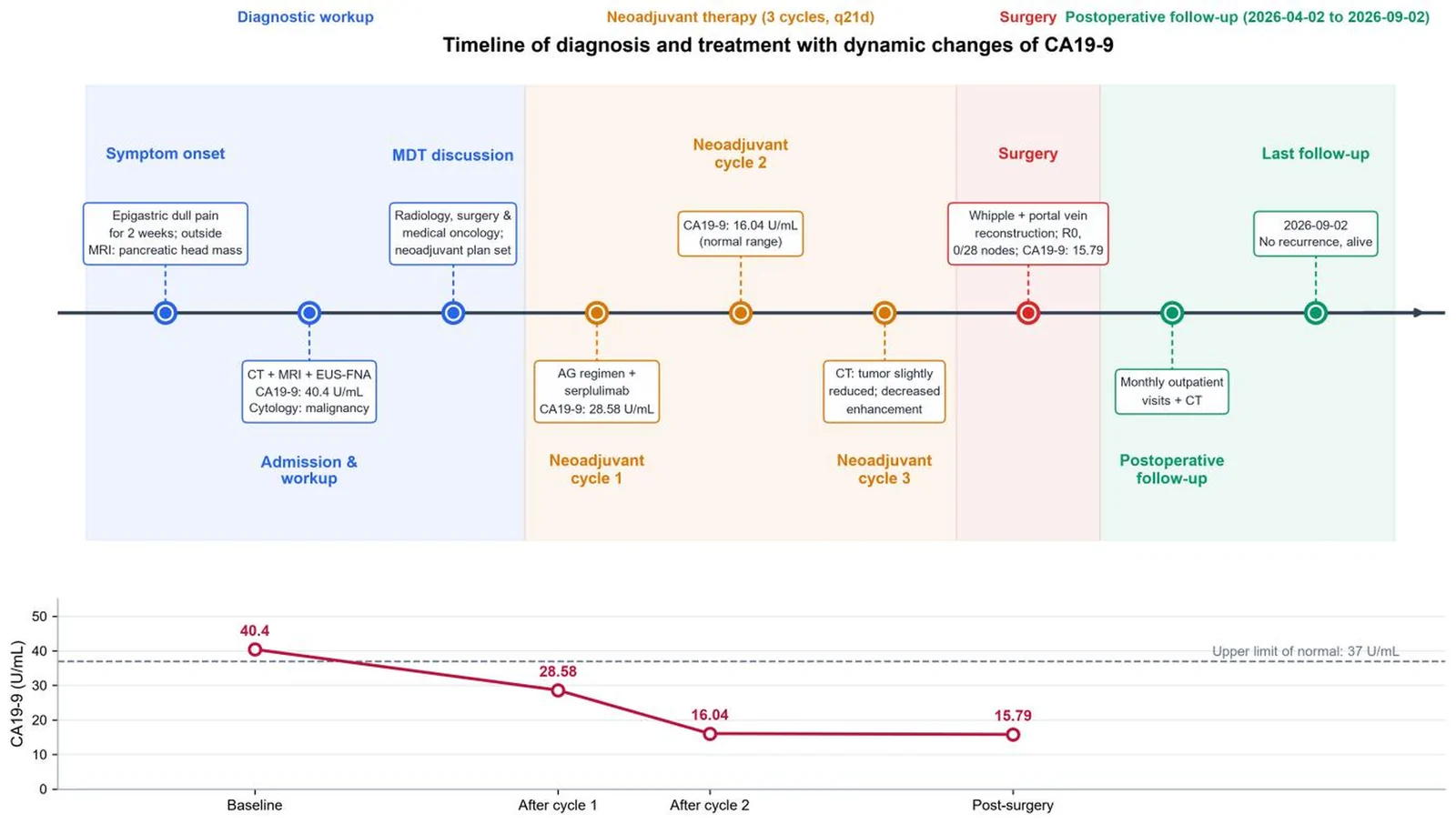
Diagnostic and treatment timeline with the dynamics of serum CA19-9 from April 2, 2026 to September 2, 2026. Imaging and EUS, multidisciplinary discussion, three neoadjuvant cycles, pancreaticoduodenectomy with portal vein resection, and serial follow-up CT are indicated along the timeline; CA19-9 values (U/mL) are plotted at baseline (40.4), after cycles 1 (28.58) and 2 (16.04), and after surgery (15.79). CT = computed tomography; EUS = endoscopic ultrasonography.

## Discussion

Primary squamous cell carcinoma (SCC) of the pancreas is an exceptionally rare malignant epithelial tumor, accounting for less than 1% of pancreatic malignancies, and its biological behavior remains incompletely understood because most available evidence is derived from isolated case reports and small retrospective series (1, 2). Normal pancreatic parenchyma lacks native squamous epithelium, and several mechanisms have therefore been proposed for its histogenesis, including malignant transformation of squamous metaplasia, differentiation of pluripotent progenitor cells, and squamous transformation within a pre-existing adenocarcinoma (1, 2). Regardless of its origin, pancreatic SCC appears to behave aggressively, with a high frequency of locally advanced disease at presentation and substantially poorer survival than conventional pancreatic ductal adenocarcinoma (PDAC) in population-based analyses (1, 2). These characteristics make early recognition and appropriate treatment planning particularly important.

Clinically, pancreatic SCC does not appear to produce a distinctive symptom complex, and most patients present with manifestations similar to those of other pancreatic malignancies, such as abdominal pain, weight loss, anorexia, or obstructive symptoms depending on tumor location (1, 3). Because of this nonspecific presentation, diagnosis is often delayed until the tumor has already reached a large size or invaded adjacent structures (1, 3). Published cases have frequently described extensive vascular involvement at diagnosis, supporting the tendency of pancreatic SCC toward aggressive local invasion (3, 4). Our patient similarly presented with a large pancreatic head-neck mass abutting the celiac axis with contact to the common hepatic and splenic arteries and encasing the main portal vein, although the superior mesenteric artery remained uninvolved and no distant metastasis was identified. This combination of extensive local vascular invasion without metastatic disease created a potential anatomical window for conversion therapy if a sufficient treatment response could be achieved.

Imaging plays an important role in suggesting this unusual histologic subtype, although no pathognomonic feature has yet been established. Previous reports have described pancreatic SCC as a relatively large cystic-solid or necrotic mass with peripheral or rim-like enhancement and frequent vascular involvement (3, 4). Similar enhancement patterns have also been reported in pancreatic adenosquamous carcinoma, in which peripheral enhancement surrounding central necrosis has been proposed as a useful feature for distinguishing tumors with squamous differentiation from conventional PDAC (5, 6). Our patient showed a highly concordant pattern, with approximately 70% central necrosis and peripheral solid tissue showing marked enhancement from 32 HU on unenhanced CT to 108 HU in the arterial phase and 117 HU in the portal venous phase. Although the lesion was initially reported as a possible cystic neoplasm with malignant transformation, the imaging findings are more consistent with a predominantly solid tumor showing central cystic degeneration: the mass had a well-defined, smooth margin with rim-like enhancement, restricted diffusion in the solid component and no thin-walled cystic structure. The differential diagnosis therefore included a solid pancreatic tumor with cystic degeneration—neuroendocrine neoplasm, solid pseudopapillary neoplasm, acinar cell carcinoma or undifferentiated carcinoma with osteoclast-like giant cells.

Diffusion-weighted imaging may provide additional information when morphologic findings are atypical. Reported apparent diffusion coefficient (ADC) values of PDAC generally fall in the range of approximately 1.0–1.3 × 10⁻^3^ mm^2^/s, although substantial overlap exists depending on technique and tumor composition (7, 8). Diffusion data for primary pancreatic SCC are extremely limited, with only isolated reports describing restricted diffusion comparable to that of other highly cellular tumors (9). In our patient, the solid component showed an ADC of 0.871 × 10⁻^3^ mm^2^/s, lower than that of the adjacent normal pancreatic parenchyma (1.173 × 10⁻^3^ mm^2^/s) and lower than many published PDAC values (7–9). A similar inverse relationship between ADC and tumor cellularity or poor differentiation has been demonstrated in squamous carcinomas of other organs, including the esophagus (10). Although a single case cannot establish a characteristic diffusion phenotype, and none of these features is specific for SCC, the combination of a well-defined margin, extensive internal necrosis, marked peripheral enhancement, restricted diffusion, and vascular involvement may raise the possibility of a pancreatic carcinoma with squamous differentiation.

Definitive diagnosis ultimately depends on histopathologic confirmation and exclusion of an extrapancreatic primary SCC. Immunohistochemical expression of squamous markers such as p40 and p63 supports squamous differentiation, whereas clinical and imaging evaluation is required to exclude metastasis from more common primary sites, including the lung, esophagus, head and neck, and cervix (1, 2). Our resected specimen demonstrated poorly differentiated SCC with positive p40 and p63 expression, CK7 negative, and no alternative primary site was identified, supporting the diagnosis of primary pancreatic SCC. the patient had no smoking history or symptoms referable to the head and neck, lung, oesophagus or gynaecological tract, and no suspicious lesion was identified on preoperative chest and abdominal imaging.

Treatment of pancreatic SCC remains challenging because no disease-specific therapeutic standard has been established, and most systemic regimens are extrapolated from PDAC (1, 2). For advanced PDAC, FOLFIRINOX and gemcitabine plus nab-paclitaxel are established first-line regimens and therefore represent reasonable therapeutic backbones for pancreatic SCC in the absence of prospective evidence (11, 12). Several published cases nevertheless suggest that pancreatic tumors with squamous differentiation may be particularly sensitive to cytotoxic therapy; complete pathological response has been reported after gemcitabine, cisplatin, and nab-paclitaxel, while conversion to surgical resection has also been achieved in unresectable pancreatic adenosquamous carcinoma after gemcitabine plus nab-paclitaxel (13, 14). These observations provide a rationale for attempting induction systemic therapy in patients with locally advanced pancreatic SCC when surgical resection is not initially feasible.

The role of immune checkpoint inhibition is even less clearly defined. Conventional PDAC is generally considered immunologically resistant, and immune checkpoint inhibitors have shown limited activity except in selected biomarker-defined subgroups (15, 16). However, isolated pancreatic SCC cases suggest that some tumors may be more immunoresponsive; Yang et al. reported a pancreatic SCC with approximately 90% PD-L1 expression that achieved a partial response to first-line sintilimab plus gemcitabine, while another highly mutated tumor showed a marked response to gemcitabine, nab-paclitaxel, and pembrolizumab (17, 18). It should be emphasised that both of those tumours harboured a strong predictive biomarker—approximately 90% PD-L1 expression in one and a high tumour mutational burden in the other—whereas no such biomarker was available in our patient. In metastatic PDAC, serplulimab combined with gemcitabine, nab-paclitaxel, and stereotactic body radiotherapy has also shown encouraging activity in early-phase investigation, supporting further exploration of PD-1-based combination strategies (15, 16). Against this background, our patient received three cycles of gemcitabine/nab-paclitaxel plus serplulimab, resulting in progressive normalization of CA19-9, substantial tumor shrinkage, and decreased enhancement on imaging. To the best of our knowledge, this appears to represent the first reported case of initially unresectable primary pancreatic SCC converted to radical resection after PD-1-based chemoimmunotherapy, although this remains an observation in a single patient and does not by itself demonstrate efficacy of PD-1 blockade.

The surgical outcome further supports the potential clinical relevance of this response. Although major vascular involvement historically limited resection in pancreatic malignancy, portal or superior mesenteric vein resection with reconstruction is now routinely considered in selected patients when an R0 resection is technically achievable (19, 20). Our patient initially had encasement of the main portal vein together with contact of the celiac axis and its branches, features consistent with locally advanced and initially unresectable disease. Following systemic therapy, pancreaticoduodenectomy with segmental portal vein resection and end-to-end portal vein reconstruction was successfully performed, resulting in microscopically margin-negative resection. The absence of tumor in all 28 retrieved lymph nodes further suggested a substantial treatment effect, although nodal negativity alone cannot prove treatment-induced downstaging. This case therefore illustrates how effective systemic therapy may create an opportunity for conversion surgery even in a pancreatic SCC with extensive initial vascular involvement.

Tumor markers may also provide useful complementary information during treatment, although their role in pancreatic SCC is not well defined. CA19-9 is the most widely used biomarker in PDAC and its serum concentration can correlate with treatment response, but it is not specific for adenocarcinoma and is also elevated in primary pancreatic SCC, as in the present case (21). SCC antigen is more closely associated with squamous differentiation and may be elevated in pancreatic SCC even when CA19-9 and CEA are normal (22). In our patient, CA19-9 declined progressively from 40.4 U/mL to within the normal range during treatment and remained normal after surgery, paralleling the radiologic and surgical response. By contrast, CA125 increased postoperatively to 82.5 U/mL despite the absence of radiologic recurrence, which may reflect postoperative inflammatory or serosal changes rather than residual malignancy. SCC antigen was unfortunately not measured, and future reports should consider incorporating SCC antigen together with CA19-9 to determine whether discordant biomarker patterns may aid diagnosis or response assessment.

The prognosis of pancreatic SCC is generally poor, particularly in unresectable disease, whereas surgical resection remains the factor most consistently associated with prolonged survival in retrospective series (1, 2). Population-based analyses have reported median survival of only a few months for unselected patients but substantially longer survival among those able to undergo curative-intent resection (1). Our patient achieved R0 resection with ypN0 disease and remained recurrence-free five months after surgery, providing encouraging early evidence of disease control. Nevertheless, the short follow-up period precludes conclusions regarding durable oncologic benefit, particularly given the aggressive natural history of pancreatic SCC.

Several limitations should therefore be acknowledged. First, PD-L1 expression, mismatch-repair/microsatellite-instability status, tumor mutational burden, and comprehensive genomic profiling were not available at the time of treatment; serplulimab was therefore given empirically and off-label, without biomarker guidance, and PD-L1 could not be assessed on the diagnostic aspirate because no cell block suitable for immunohistochemistry was obtained. Second, because gemcitabine plus nab-paclitaxel alone can induce tumour regression and downstaging in locally advanced pancreatic cancer, the contribution of PD-1 blockade to the response observed here cannot be distinguished from that of the chemotherapy backbone, and the possibility that serplulimab provided no additional benefit cannot be excluded. Third, SCC antigen was not measured (17, 22), and follow-up remains short. This case therefore provides no evidence of efficacy for PD-1-based chemoimmunotherapy, and the regimen used here should not be adopted outside a clinical trial. It does suggest, however, that SCC, although rare, should be included in the differential diagnosis of solid pancreatic malignancies with internal necrosis, and that prospective evaluation of PD-1-based chemoimmunotherapy—with mandatory biomarker profiling for PD-L1, MSI/MMR and tumour mutational burden—is warranted before any clinical recommendation can be made. Finally, the entire resected specimen was sampled at multiple cuts and no glandular component or mucin was identified.

## Patient perspective

The patient reported that her abdominal pain resolved after the operation and that she returned to her usual daily activities without restrictions at the last follow-up in early September 2026.

## Data Availability

The raw data supporting the conclusions of this article will be made available by the authors, without undue reservation.
